# Non-linear interactions in cosmologies with energy exchange

**DOI:** 10.1140/epjc/s10052-020-7642-2

**Published:** 2020-02-13

**Authors:** John D. Barrow, Georgia Kittou

**Affiliations:** 1 DAMTP, Centre for Mathematical Sciences, University of Cambridge, Wilberforce Rd., Cambridge, CB3 0WA UK; 2 College of Engineering and Technology, American University of the Middle East, Egaila, Kuwait

## Abstract

We investigate the case of two interacting fluids in homogeneous and isotropic cosmologies with a non-linear interaction term. The interaction term avoids the unrealistic form generally used in the literature, beginning with Tolman, in which the interaction is zero when the Hubble parameter vanishes. A variety of exact solutions for the scale factor are found and describe a range of new behaviors. We also extend the analysis of possible cosmological solutions with interacting fluids when curvature is taken into consideration. We use an example of energy exchange between radiation and scalar field to alleviate the flatness problem.

## Introduction

In this study, we consider cosmological models that can describe the observed acceleration of the universe, by modifying the standard picture of the material content of the universe. We introduce a multi-fluid universe with a mutual energy exchange between the fluid components, in such a way that the total energy is conserved [1–3]. Assuming that one of the fluids belongs to the ’dark sector’, then the cosmic acceleration can be caused by the late dominance of this fluid.

The study of energy exchange between cosmological fluids is quite extensive because of its wide applicability. Many physical processes can be modelled by interacting fluids: particle-anti particle annihilation into radiation [4, 5], the process of black-hole formation and quantum evaporation [6–8], and the decay of other forms of mass-energy into radiation. A list of specific interaction functions can be found in literature, see [9]. The interaction function where energy exchange is proportional to the product of the Hubble expansion rate times an arbitrary linear combination of energy densities of the two fluids appears by Barrow and Clifton [9] and encompasses many of these applications. An interaction term proportional to the product of energy densities of dark matter and dark energy was introduced in Refs. [10, 11]. This interaction function is widely used in ecology to describe prey-predator models. In cosmology such an interaction term is used as a way-out of the coincidence problem [12, 13]. A more general ansatz for the interaction is used in the so-called Chaplygin gas model [11] to present a unified dark matter and dark energy model with the first fluid dominating at early times and the other dominating at late times. By introducing an energy exchange between scalar and matter fields in scalar tensor theories, one can also examine the erosion of the value of the gravitational ‘constant’ *G* over cosmological timescales.

When non-zero curvature is included in the field equations, the behavior of the scale factor changes significantly at large expansion times. For positively curved universes, oscillatory solutions can be obtained. In cases of spatial flatness, it was shown in [14] that oscillatory behavior can be produced when one of the fluid components has a negative (‘ghost’) vacuum energy. In Ref. [15] a fuller description shows how exact solutions for energy exchange can describe oscillating closed universes with constant and increasing entropy in cosmologies with both varying, and unchanging physical, constants.

The plan of the paper is as follows. In Sect. 2 we introduce a new interaction term where the Hubble parameter is not contained explicitly and explain why this is physically more realistic than previous models. In Sect. 3 we write down the field equations and arrive in an autonomous master equation related to the Hubble expansion. Then, two different approaches are followed to obtain exact solutions for the expansion of the scale factor. In Sect. 4 the effects of spatial curvature are included in the field equations and in the last section we summarise our results.

## The interaction term

Consider a flat Friedmann–Robertson–Walker (FRW) universe containing two cosmological fluids $\rho _{1},\rho _{2}$ that are exchanging energy in such a way that the total energy is conserved. We introduce an interaction term, *Q*, so that the individual equations of energy conservation for both fluids are given respectively by

1 $$\begin{aligned} \dot{\rho _{1}}+3H\Gamma \rho _{1}= & {} Q, \end{aligned}$$

2 $$\begin{aligned} \dot{\rho _{2}}+3H\gamma \rho _{2}= & {} -Q, \end{aligned}$$

and the total density is conserved. Here, Γ and γ are the barotropic indices for each fluid,

$$\begin{aligned} p_{1}=(\Gamma -1)\rho _{1}\quad \text {and }\quad p_{2}=(\gamma -1)\rho _{2}, \end{aligned}$$

and $H=\dot{a}/a$ is the Hubble parameter; *a*(*t*) expansion scale factor of the universe and *t* is comoving proper time.

An energy-conserving exchange model of this sort was first introduced by Tolman [16] in 1934 to describe the transfer of energy from matter ($\rho _{2}$) to blackbody radiation ($\rho _{1}$) in the universe – ‘from the nebulae into internebular space’ as he described it. Tolman chose $Q=\gamma H\rho _{2}$, where γ>0 is a (small) constant ($\gamma \simeq 10^{-7}-10^{-4}$), so a first integral is found to be $\rho _{2}\propto a^{-3-\gamma }$.^1^

From a dimensional point of view it is expected that the interaction term, *Q*, should be a function of the Hubble parameter and energy density [6, 16, 17]. In [9], the authors considered an interaction term of the form $Q=H(-\beta \rho _{1}+\alpha \rho _{2})$ to describe a mutual energy exchange between two cosmological fluids. Their choice complies with the current observational data. In particular, to alleviate the coincidence problem [18, 19], an interaction between dark matter and dark energy is taken into consideration, and it is shown that for such interaction term the energy densities $\rho _{1}$ and $\rho _{2}$ evolve at the same rate.

Almost all past studies of interacting fluids, from Tolman onwards, have assumed $Q\propto H\rho$. This is an obvious mathematical reason to do this: it allows for an immediate integration of (1) or (2) with respect to *t*. Hence, the specific form of interaction is used for mathematical simplicity in order to obtain analytical solutions. However, this form for the interaction term it not physically very reasonable: it implies that the energy exchange becomes zero when H=0. This property seems to be entirely unrealistic since the energy exchange is mediated by local physical processes. There is no reason why they should switch off if the expansion of the universe momentarily halts everywhere at the expansion maximum of a closed universe, or in large over-densities that reach an expansion maximum before collapsing under their local self-gravity. Therefore, in what follows, we will not make the usual (unrealistic) assumption that Q∝H. Instead, we propose a new interaction term *Q* of the form

3 $$\begin{aligned} Q=\frac{1}{t}(-\beta \rho _{1}+\alpha \rho _{2}), \end{aligned}$$

where *t* is the cosmic time. The interaction term is a leading order in a series expansion of possible contributions and allows us to build up intuition about the consequences of this type of interaction. It is shown in [20] that the interaction function is generally dependent on the scale factor under the form $Q\sim a^{-3}$. Consequently, the new interaction term is consistent with respect to dimensional analysis. The energy-exchange parameters, are taken to be α and β, are real and positive, without loss of generality.

A comment regarding the magnitude of the energy-exchange parameters α and β is in order. If $\alpha =\beta$, then the strength of coupling to the geometry of the 3-slice is taken to be the same for both fluids. In general, the interaction term (3) describes a ‘one-way’ interaction where the first fluid $\rho _{1}$ decays, whereas the second fluid is gaining energy equal to that lost by the first fluid. This occurs at early times where $\rho _{1}>\rho _{2}$. Then, at late times ($\rho _{2}>\rho _{1}$), the interaction term changes sign as the second fluid now decays and the first fluid gains energy. Generally, at early times, a ‘one-way’ interaction is feasible if $\frac{1}{t}(-\beta \rho _{1}+\alpha \rho _{2})<0$ with $\rho _{1}>\rho _{2}$. Similarly, at late times, if $\frac{1}{t}(-\beta \rho _{1}+\alpha \rho _{2})>0,$ where $\rho _{2}>\rho _{1}$.

In the early universe it is expected that density perturbations in the matter give rise to the overdensities needed for the formation of galaxies [21]. Therefore, by assuming an interaction between, say, dark matter ($\rho _{1}$) and dark energy ($\rho _{2}$), it is expected that the interaction term will be negative since $\rho _{1}>\rho _{2}$. Now, as the expansion of the universe changes from decelerated to accelerated, the sign [22–24] of the interaction term (3) will become positive in a natural way since $\rho _{2}>\rho _{1}$. We emphasize here that our new interaction term (3) is consistent with the second law of thermodynamics [23],

4 $$\begin{aligned} \dot{S_{1}}+\dot{S_{2}}=\left( \frac{1}{T_{1}}-\frac{1}{T_{2}}\right) Q\ge 0, \end{aligned}$$

where $$\dot{S_{i}}$$ represents the change of entropy of each fluid in time, $$T_{i}$$ is the temperature for each fluid and *Q* the energy exchange. We expect the entropy of the universe to be non-decreasing with time but we do not yet know all the repositories of entropy in the Universe. Equation (4) implies that as the universe expands, the temperature of $$\rho _{1}$$ (assume dark matter) decreases while the temperature of $$\rho _{2}$$ (assume dark energy) increases. Since at early times $$\rho _{1}>\rho _{2}$$ (that is Q<0) and $$T_{1}>T_{2}$$, the total entropy of the system is positive. In addition, the law (4) is satisfied at late times and the universe undergoes an accelerated expansion, since $$\rho _{2}>\rho _{1}$$ (that is Q>0) and $$ T_{2}>T_{1}$$.

## The master equation

In this section we derive a master equation that governs the evolution of the Hubble parameter *H* for a system of two mutually interacting fluids in a flat FRW universe, by means of the Friedmann equation (units are chosen where 8πG=c=1)

5 $$\begin{aligned} 3H^{2}=\rho _{1}+\rho _{2}, \end{aligned}$$

and the continuity equations for both fluids

6 $$\begin{aligned} \dot{\rho _{1}}+3H\Gamma \rho _{1}= & {} \frac{1}{t}(-\beta \rho _{1}+\alpha \rho _{2}), \end{aligned}$$

7 $$\begin{aligned} \dot{\rho _{2}}+3H\gamma \rho _{2}= & {} \frac{1}{t}(\beta \rho _{1}-\alpha \rho _{2}), \end{aligned}$$

respectively. Using the last three equations, we can eliminate the energy densities to obtain a single non-autonomous master equation for the Hubble expansion

8 $$\begin{aligned} \ddot{H}+ & {} \dot{H}\left[ \frac{1}{t}(\alpha +\beta )+3H(\Gamma +\gamma )\right] \nonumber \\+ & {} \frac{3H^{2}}{2t}(\alpha \Gamma +\beta \gamma ) +\frac{9}{2}H^{3}\gamma \Gamma =0. \end{aligned}$$

Under the transformation H=u/t where $t=e^{x}$, the equation above reduces to an autonomous, second-order non-linear differential equation of the form

9 $$\begin{aligned} u{\prime \prime }+Au{\prime }+Buu{\prime }-(A+1)u+(C-B)u^2+Du^3=0,\nonumber \\ \end{aligned}$$

where prime denotes differentiation with respect to *x* and

10 $$\begin{aligned} A= & {} (\alpha +\beta -3),\quad B=3(\Gamma +\gamma ),\nonumber \\ C= & {} \frac{3}{2} (\beta \gamma +\alpha \Gamma ),\quad D=\frac{9}{2}\Gamma \gamma . \end{aligned}$$

Equation (9) is considered to be a Liénard type ordinary non-linear differential equation [25, 26]. In what follows, we rewrite Eq. (9) in the compact form

11 $$\begin{aligned} u^{\prime \prime }+f(u)u^{\prime }+g(u)=0, \end{aligned}$$

where f(u)=A+Bu and $g(u)=-(A+1)u+(C-B)u^2+Du^3$. It is argued in [25, 26] that if the function *g*(*u*) satisfies the condition

12 $$\begin{aligned} g(u)=f(u)\left[ c_1+k\int {f(u)du}\right] , \end{aligned}$$

where $c_{1}$ and *k* are arbitrary constants, then Eq. (11) is exactly integrable. After some manipulations, we find that this integrability condition is satisfied for different pair of values for the constants $c_{1}$ and *k*, as well as the parameters *A*, *B*, *C*,  and *D*.

### Case I

For the first case, condition (12) is satisfied for

13 $$\begin{aligned} c_1=0,\quad k=-\frac{A+1}{A^2},\quad C-B=\frac{3}{2}kAB,\quad D=\frac{k}{2} B^2. \end{aligned}$$

The function *f*(*u*) is given by f(u)=A+Bu, where the constants *A*, *B* are defined above. Then, from the integrability condition (12), we obtain the function *g*(*u*) as

14 $$\begin{aligned} g(u)=kA^{2}u+\frac{3}{2}kBu^{2}+\frac{1}{2}kB^{2}u^{3}. \end{aligned}$$

The exactly integrable Liénard equation is now given by

15 $$\begin{aligned} u^{\prime \prime }+(A+Bu)u^{\prime }+\left( kA^{2}u+\frac{3}{2}kBu^{2}+\frac{ 1}{2}kB^{2}u^{3}\right) =0.\nonumber \\ \end{aligned}$$

The equation above with f(u)=A(u)+B(u)u was first used by Dumortier and Rousseau^2^ in [36] to describe the case of linearly forced isotropic turbulence. Following the analysis expounded in [25, 26], the non-linear equation (15) has a general solution in a parametric form and satisfies the equation

16 $$\begin{aligned} \frac{g(u)}{f(u)}=\tilde{C}^{-1}\exp [F(w,k)], \end{aligned}$$

where $\tilde{C}^{-1}$ is a constant of integration and exp[F(w,k)] is defined in [25]. The time dependence of *u* is determined as a function of *w* by

17 $$\begin{aligned} x-x_{0}=\int \frac{dw}{f(u(w))(w^{2}+w+k)}. \end{aligned}$$

We note that Eq. (16) is equivalent to the quadratic form

18 $$\begin{aligned} Au+\frac{B}{2}u^{2}=\frac{\tilde{C}^{-1}}{k}\exp [F(w,k)], \end{aligned}$$

and has real solutions for k<1/4. An exact solution of the equation above is

19 $$\begin{aligned} u(w)=\frac{-A\pm \sqrt{A^{2}+2B\tilde{C}^{-1}\exp [F(w,k)]/k}}{B}, \end{aligned}$$

where exp[F(w,k)] has a special form for k<1/4 (see Eq. (17) in [25]).

The exact parametric solutions also allow us to obtain some approximate solutions of the differential equation (15). Assuming w<<k (early time asymptote), Eq. (18) takes the form

20 $$\begin{aligned} \frac{B}{2}u^{2}\approx \frac{\tilde{C}^{-1}w}{k}, \end{aligned}$$

and the parametric time evolution satisfies

21 $$\begin{aligned} x-x_{0}\approx \frac{1}{k}\int {\frac{dw}{f(u(w))}}. \end{aligned}$$

After some manipulations we find that the asymptotic solution at early times is

22 $$\begin{aligned} u^{2}=\frac{2}{k}(\exp {[B\tilde{C}^{-1}(x-x_{0})]}-A^{2}), \end{aligned}$$

which gives time-evolution of the scale factor:

23 $$\begin{aligned} a(t)\sim \exp {\left( t^{B\tilde{C}^{-1}/2}\right) }. \end{aligned}$$

The specific form of Eq. (23) describes a time-evolution of the universe, with slower rate of expansion than the de Sitter universe [28–30] called intermediate inflation [31, 32] when $B\tilde{C}^{-1}/2<1$. If $B\tilde{C}^{-1}/2>2$ then there would be a future curvature singularity. We note here that both fluids dictate the asymptotic solution since $B=3(\Gamma +\gamma )$.

In the limit of large *w*, so that w>>k and $w^{2}>>w$ (at late times), we get the asymptotic form $\exp [F(w,k)]\approx \exp {(-k/(2w^{2}))}$, and

24 $$\begin{aligned} \frac{B}{2}u^{2}\approx \frac{\tilde{C^{-1}}}{k}\exp {\left( -\frac{k}{2w^{2} }\right) }. \end{aligned}$$

The parametric time-evolution is

25 $$\begin{aligned} x-x_{0}\approx \int {\frac{dw}{f(u(w))}w^{2}}. \end{aligned}$$

Following the same steps as above, we get the asymptotic solution

26 $$\begin{aligned} \frac{B}{2}u^{2}=\frac{\tilde{C^{-1}}}{k}\exp {\left[ \frac{x(x-x_{0})^{2}}{ 2(A^{2}+2B\tilde{C^{-1}/k})}\right] }, \end{aligned}$$

or, in terms of the scale factor,

27 $$\begin{aligned} a(t)\sim \exp {\left( t^{\xi }\right) }, \end{aligned}$$

where

28 $$\begin{aligned} \xi =\frac{k}{A^2+2B/(\tilde{C}k)}. \end{aligned}$$

The asymptotic form of the solution (27) is of interest since it can describe the case of decelerated expansion throughout cosmological evolution (since k<1/4).

### Case II

For the second case, condition (12) is satisfied for

29 $$\begin{aligned} c_1=-\frac{1}{B},\quad k=\frac{2D}{B^2},\quad A=0,\quad C=B. \end{aligned}$$

The equation above imposes some physical restrictions on the energy exchange parameters and well as the barotropic indices of the fluids. Specifically, for A=0 it occurs that $\alpha +\beta =3$, hence the strength of coupling for each fluid with the geometry of the 3-slice is bounded. Also, for C=B, we find that $\alpha =(2\Gamma -\gamma )/(\Gamma -\gamma )$. Since α is taken to be positive the barotropic indices must satisfy the condition

30 $$\begin{aligned} \Gamma >\gamma ,\quad \text {or}\quad \Gamma <\frac{1}{2}\gamma . \end{aligned}$$

Assuming that the second fluid satisfies γ=0, then second inequality opens up the possibility for the first interacting fluid being a ‘phantom’ [33, 34] satisfying Γ<0.

To find the exact solutions for the scale factor, we follow a different approach to that for Case I. Taking into consideration the analysis used in [27], we conclude that the differential equation

31 $$\begin{aligned} u^{\prime \prime }+Buu^{\prime }-u+Du^3=0. \end{aligned}$$

has the following invariant form,

32 $$\begin{aligned} \bar{u}^{\prime \prime }+\bar{u}^{\prime }+\frac{2\bar{D}}{\bar{B}^{2}}\bar{u }-\frac{1}{\bar{B}}=0, \end{aligned}$$

where $\bar{u}=\int f(u)du$ and $\bar{x}=\int f(u)dx$. For the homogeneous part of the linear equation above, the roots of the characteristic equation are

33 $$\begin{aligned} \lambda _{1,2}=\frac{-1\pm \sqrt{1-4\bar{k}}}{2}, \end{aligned}$$

with $\bar{k}=2\bar{D}/\bar{B^2}$. A real solution of the form

34 $$\begin{aligned} \bar{u}(\bar{x})=c_1\exp ({\lambda _1 \bar{x}})+c_2\exp ({\lambda _2 \bar{x}})+ \frac{\bar{B}}{2\bar{D}}, \end{aligned}$$

is obtained for $\bar{k}<1/4$. From (29) it follows that the solution above describes the case of decaying fluids with $\Gamma >\gamma$. We note here that for $\bar{k}=1/4$ the problem is reduced to one-fluid description with no interaction since then $\Gamma =\gamma$.

The transformation of variables $\bar{u}=\int f(u)du$ and $\bar{x}=\int f(u)dx$ relates the general solution of Eqs. (11)–(32) through the transformation $\bar{y}(\bar{x})$. After some calculations, we find that the general solution of (11) has the form

35 $$\begin{aligned} u^{2}(x)=a^{-B/2}\left[ c_{3}a^{\sqrt{B^{2}-8D}/2}+c_{4}a^{-\sqrt{B^{2}-8D} /2}\right] +\frac{1}{D}, \end{aligned}$$

where *a* is the scale factor and $c_{3},c_{4}$ are constants. For simplicity, let us assume that the constant term in the equation above is negligible. It follows that at early times, as a→0, we then have

36 $$\begin{aligned} u^{2}(x)\rightarrow a^{-(B+\sqrt{B^{2}-8D})/2}, \end{aligned}$$

whereas, at late times where $a\rightarrow \infty$,

37 $$\begin{aligned} u^{2}(x)\rightarrow a^{(-B+\sqrt{B^{2}-8D})/2}. \end{aligned}$$

These two equations can be integrated to obtain the following exact solution for the scale factor

38 $$\begin{aligned} a_{\pm }\propto (lnt)^{4/(B\pm \sqrt{B^{2}-8D})} \end{aligned}$$

at early (+branch) and late times (−branch) respectively. Now, inserting (10) into the latter solution we get the simplified forms

39 $$\begin{aligned} a\propto (lnt)^{2/(3\Gamma )},\quad \text { as }\quad a\rightarrow 0, \end{aligned}$$

and

40 $$\begin{aligned} a\propto (lnt)^{2/(3\gamma )},\quad \text { as }\quad a\rightarrow \infty . \end{aligned}$$

The specific form of time evolution for the scale factor $a=(lnt)^{\xi }$ was previously found as one of the seven ever-expanding possibilities in [37]. Here, this specific type of solution describes an expanding but non-inflationary universe for ξ>0.

The dynamic behaviours of the solutions (39) and (40) are determined by the barotropic indices Γ and γ, respectively, and two types of evolution occur. First, assuming that both barotropic indices are positive, the universe will experience an initial singularity at a finite time $t^{*}$ and continue expanding. For the second and more interesting scenario, assuming that the barotropic indices have opposite signs [35] (without loss of generality assume Γ>0 and γ<0) then as $t\rightarrow \infty$, an inflationary expansion will occur. Specifically, the universe experiences a singularity at early times, followed by a period of decelerated expansion (since ξ>0), and at late times the dominance of phantom fluid (with ξ<0) triggers the transition to an accelerated expansion.

In recent works, [38, 39], the case of two interacting fluids in the dark sector is modelled by a unified model of a single bulk viscous fluid. There, the authors describe a transition from a phase of decelerated expansion to one of accelerated expansion under some restrictions on the value of bulk viscosity coefficient.

## Curved case scenario

In this section we will study the case of curved FRW universes filled with two interacting fluids. Our main purpose is to examine how the evolution of the scale factor and Hubble parameter respectively are changed, when we pass on to curved universes while keeping the interaction term (3) the same. Recall, that we were motivated in our choice of interaction terms, Eq. (3) by a desire to avoid the explicit presence of *H* in *Q* in order to avoid unphysical behaviour when H=0 and H<0 when an expansion maximum occurs. The Friedman equation and conservation equations now read,

41 $$\begin{aligned} 3H^{2}+\frac{3k}{a^{2}}= & {} \rho _{1}+\rho _{2}, \end{aligned}$$

42 $$\begin{aligned} \dot{\rho _{1}}+3H\Gamma \rho _{1}= & {} Q, \end{aligned}$$

43 $$\begin{aligned} \dot{\rho _{2}}+3H\gamma \rho _{2}= & {} -Q, \end{aligned}$$

where the curvature parameter k=±1, *a* is the scale factor and the interaction term still has the form $Q=\frac{1}{t}(-\beta \rho _{1}+\alpha \rho _{2})$.

Now, we seek exact solutions for the scale factor. First, we take the time derivative of Eq. (5) and after manipulations we find the evolution equation for the Hubble parameter *H*(*t*) is,

44 $$\begin{aligned} \dot{H}+\frac{3}{2}\gamma H^{2}=\frac{1}{2}(\gamma -\Gamma )\frac{m}{ a^{3\Gamma }}+\frac{k}{a^{2}}(1-3\gamma /2). \end{aligned}$$

Here, we have used the relation [40]

45 $$\begin{aligned} \rho _{1}=\frac{\int {Qa^{3\Gamma }}dt}{a^{3\Gamma }}\equiv \frac{m}{ a^{3\Gamma }}, \end{aligned}$$

which occurs after integrating Eq. (41), and

$$\begin{aligned} m(t)\equiv \int {Qa^{3\Gamma }}dt. \end{aligned}$$

In terms of the scale factor the evolution equation (44) reads

46 $$\begin{aligned} \frac{\ddot{a}}{a}-\frac{1}{a^{2}}(1-3\gamma /2)(k+\dot{a}^{2})=\frac{1}{2} (\gamma -\Gamma )\frac{m(t)}{a^{3\Gamma }}. \end{aligned}$$

Using the conformal time transformation adη=dt and new variable [41] $b=a^{3\Gamma -2}$, it can be shown that the equation above is equivalent to

47 $$\begin{aligned} b^{\prime \prime }+\frac{k}{2}(2-3\gamma )(2-3\Gamma )b=\frac{1}{2} (2-3\Gamma )(\Gamma -\gamma )m(\eta ), \nonumber \\ \end{aligned}$$

where $^{\prime }\equiv d/d\eta$, which in turn describes the equation of motion for a forced harmonic oscillator subject to a time-dependent force for Q≠0. We note here that this equation is valid under the constraint $6\Gamma =2+3\gamma$, which restricts the possible type of cosmological fluids used.

The solution to the associated homogeneous equation reads

48 $$\begin{aligned} b_{c}(\eta )= & {} c_{1}\cos \left[ \sqrt{(2-3\Gamma )(2-3\gamma )k/2}\eta \right] \nonumber \\&+\,c_{2}\sin \left[ \sqrt{(2-3\Gamma )(2-3\gamma )k/2}\eta \right] , \end{aligned}$$

where $\omega _{0}^{2}=(2-3\gamma )(2-3\Gamma )k/2$ is the natural frequency of oscillations and depends on the barotropic indices of the interacting fluids and the spatial curvature. Any general time-dependent periodic function can be decomposed into its Fourier cosine and sine components. Assuming that the external force $F_{e}(\eta )$ given by the right-hand side of Eq. (47) is periodic, the particular solution of the associated problem reads,

$$\begin{aligned} b_{p}(\eta )= & {} \frac{\gamma -\Gamma }{k(3\gamma -2)}\cos \left[ \sqrt{(2-3\Gamma )(2-3\gamma )k/2}\eta \right] \\&\times \int {m(\eta )\sin \left[ \sqrt{(2-3\Gamma )(2-3\gamma )k/2}\eta \right] d\eta } \\&+\frac{\gamma -\Gamma }{k(3\gamma -2)}\sin \left[ \sqrt{(2-3\Gamma )(2-3\gamma )k/2}\eta \right] \\&\times \int {m(\eta )\cos \left[ \sqrt{(2-3\Gamma )(2-3\gamma )k/2}\eta \right] d\eta }. \nonumber \end{aligned}$$

The general solution to Eq. (47) is a combination of the solution for the homogeneous part of the equation, $b_{c}$ and the particular solution, $b_{p}$. Thus,

$$\begin{aligned} b(\eta )= & {} c_{1}\cos \left[ \sqrt{(2-3\Gamma )(2-3\gamma )k/2}\eta \right] \\&+c_{2}\sin \left[ \sqrt{(2-3\Gamma )(2-3\gamma )k/2}\eta \right] \\&+\frac{\gamma -\Gamma }{k(3\gamma -2)}\cos \left[ \sqrt{(2-3\Gamma )(2-3\gamma )k/2}\eta \right] \\&\times \int {m(\eta )\sin \left[ \sqrt{(2-3\Gamma )(2-3\gamma )k/2}\eta \right] d\eta } \nonumber \\&+\frac{\gamma -\Gamma }{k(3\gamma -2)}\sin \left[ \sqrt{(2-3\Gamma )(2-3\gamma )k/2}\eta \right] \\&\int {m(\eta )\cos \left[ \sqrt{(2-3\Gamma )(2-3\gamma )k/2}\eta \right] d\eta }. \end{aligned}$$

For simplicity, we assume an “one-way” interaction of the form $Q=-\beta \rho _{1}t^{-1}$^3^ so that the first fluid decays into the second fluid at a rate proportional to its energy density. As a result, the energy loss from the first fluid is gained by the second fluid and the total energy is conserved. It is shown (see Appendix in [40]) that for such interaction term, the corresponding time-dependent function *m* takes the form $m(\eta )=m_{0}e^{-\beta \eta }$, where $m_{0}$ a constant of integration. The solution for k≠0 then takes the form

49 $$\begin{aligned} b(\eta )= & {} c_{1}\cos \left[ \sqrt{(2-3\Gamma )(2-3\gamma )k/2}(\eta -\eta _{0})\right] \nonumber \\&+ \frac{(\gamma -\Gamma )m_{0}e^{-\beta \eta }}{k(3\gamma -2)+\beta ^{2}}. \end{aligned}$$

### An oscillating toy-model

Let us now examine the behavior of the exact solution found above for a positively curved universe containing radiation (Γ=4/3) and scalar field (γ=2). Both barotropic indices satisfy the constraint imposed when obtaining Eq. (47). We assume without loss of generality that the energy exchange parameter β is negative so that energy is being transferred from scalar field (that is $\rho _{2}$) to radiation ($\rho _{1}$). The exact solution in terms of the scale factor is,

50 $$\begin{aligned} a^{2}(\eta )=|c_{1}\cos {[2(\eta -\eta _{0})]}|+\frac{2m_{0}e^{\beta \eta }}{ 3(4+\beta ^{2})}. \end{aligned}$$

We use this form to avoid negative values of the scale factor and ensure that it will be real and semi-finite throughout cosmic evolution.

At early times, the oscillatory part of the solution dominates the expansion of the universe. Each successive maximum is separated by a collapse of the scale factor to zero size. Energy is being transferred from the scalar field to the radiation at a slow rate which results in the same amplitude of oscillation for each cycle. As time increases, the amplitude of each cycle increases monotonically and asymptotically the universe is pushed closer and closer to spatial flatness, with each successive cycle being longer and longer lived [14, 20, 42].

Moreover, the form of solution (50) suggests that after a number of oscillatory cycles, a collapse singularity will be avoided in the late future. Rather, a series of non-zero expansion minima will take place. This behavior occurs because at this point of evolution, the endless energy transfer of energy from scalar field to radiation results in the former obtaining negative energy density allowing non-zero expansion minima [14, 16, 40]. At late times, the universe is expected to experience a run-away behavior as the exponential term dominates the evolution. As a result the universe will expands eternally. This is characteristic of the behaviour of closed universes with a positive cosmological constant [14]. Entropy increase will increase the size of the successive expansion maxima until they become large enough for the cosmological constant to influence the dynamics. When it does, the oscillations will cease and the expansion will approach de Sitter expansion as $t\rightarrow \infty$. The final state will be dominated by the cosmological constant but lie quite close to the state where the density parameters of the cosmological constant and the matter, with a closeness determined by the size of the entropy increase from one cycle to the next. This evolution will change in some respects if anisotropies are included. Although successive cycles increase in size and entropy they also become increasingly anisotropic at their expansion volume maxima. If a cosmological constant stops these oscillations it will isotropise the expansion on approach to de Sitter as $t\rightarrow \infty$ [42, 43].

## Discussion

In this work, we have investigated both the behavior of solutions of spatially flat and curved FRW universes, with the inclusion of mutual energy exchange between two cosmological fluids. In particular, the interaction term we employ has a more realistic form than is generally employed in the literature, ensuring that the energy exchange between the interacting fluids does not cease when the Hubble parameter is zero.

The case of interacting fluids has attracted growing interest over the past years, and the idea of energy exchange between cosmological fluids has been espoused as a mean to explain recent observational data. To be more precise, the presence of a coupling between dark energy and dark matter is an attempt to alleviate the ‘coincidence problem’ between their present energy densities [44]. In addition, various physical processes such as particle-antiparticle annihilation into radiation, black hole evaporation, particle decays, and vacuum decay into radiation can be modelled as examples of interacting cosmic fluids.

Considering all these possible applications, we look for solutions for the expansion of the scale factor of FRW universes of all curvatures and none, in the presence of energy exchange between two fluid components using a non-linear interaction term. First, we considered the case of a system of two mutually interacting fluids in flat FRW universes. Using the Friedman equation and the conservation equations for both fluids we obtained a second-order, non-autonomous, non-linear differential equation describing the evolution of the Hubble parameter. Under a transformation, the master equation is reduced to a single autonomous, non-linear differential equation. The reduced differential equation is identical to an exact, integrable Li énard type equation under some restrictions. To find exact solutions to the problem, we followed two different techniques expounded in the literature.

For the first case, we use the Chiellini integrability condition to obtain a class of exact solutions of the Liénard equation expressed in a parametric form. At early times we show that the asymptotic solution for the scale factor satisfies describes a so-called phase of intermediate inflation. On the other hand, at late times the form of the scale factor shows that the universe undergoes decelerated expansion throughout cosmic evolution.

For the second case, the reduced differential equation is written under a form-invariant transformation as a second order, linear, non-homogeneous differential equation. As before, two asymptotic solutions are obtained at early and late times respectively. Both solutions found describe the case of an expanding but non-inflationary universe (assuming both barotropic indices $\gamma ,\Gamma$ are positive). In the case, if we allow barotropic indices with opposite signs, then the presence of phantom fluids drives the evolution of the universe towards an accelerated expansion.

In the last section, the case of interacting fluids in curved FRW cosmologies is examined. Under a conformal transformation and a restriction on the barotropic indices, the master equation describing the expansion of the scale factor is identical to the form of a forced harmonic oscillator (for non-zero interaction term). We have shown that the general solution governing the evolution of the universe in the case of positive spatial curvature follows an eternal series of cycles at early times. Each one of these cycles begins with a big bang singularity and ends with a big crunch singularity. After each crunch-to-bang transition, the entropy of the universe increases, As a result, the amplitude of oscillations increases progressively. A late times, the presence of the exponential term dominates the oscillatory expansion the universe and after a finite number of future cycles there are no further oscillations. Thus, at late times the universe is singular free. Finally, assuming a toy-model of one-way of interaction between radiation and scalar field, we show that, as expected at late times, the collapse singularity after each successive maximum is avoided.

## Data Availability

This manuscript has no associated data or the data will not be deposited. [Authors’ comment: This is a theoretical study and no experimental data has been listed.]

## Footnotes

1 In [16], Tolman’s g(t)=2ln(a) and his mass function is $M(t)=\frac{4\pi }{3}\rho _{m}a^{3}.$

2 Where *A*(*u*) a cubic function and *B*(*u*) a linear function.

3 Here, the interaction term is similar to the one used in [40], if we assume that the scale factor describes a Milne universe so that a=t.
